# Comparison of Single Phase and Biphasic Extraction Protocols for Lipidomic Studies Using Human Plasma

**DOI:** 10.3389/fneur.2019.00879

**Published:** 2019-08-21

**Authors:** Matthew Wai Kin Wong, Nady Braidy, Russell Pickford, Perminder Singh Sachdev, Anne Poljak

**Affiliations:** ^1^Centre for Healthy Brain Ageing, School of Psychiatry, Faculty of Medicine, University of New South Wales, Sydney, NSW, Australia; ^2^Bioanalytical Mass Spectrometry Facility, University of New South Wales, Sydney, NSW, Australia; ^3^Euroa Centre, Prince of Wales Hospital, Neuropsychiatric Institute, Sydney, NSW, Australia; ^4^School of Medical Sciences, Faculty of Medicine, University of New South Wales, Sydney, NSW, Australia

**Keywords:** lipidomics, lipid extraction, mass spectrometry, plasma lipids, chromatography

## Abstract

Lipidomic profiling of plasma is an emerging field, given the importance of lipids in major cellular pathways, and is dependent on efficient lipid extraction protocols. Recent attention has turned to plasma lipidomics as a means to identify potential diagnostic and prognostic biomarkers related to dementia, neuropsychiatric health and disease. Although several solvent-based lipid extraction protocols have been developed and are currently in use, novel and more efficient methods could greatly simplify lipid analysis in plasma and warrant investigation. Human plasma from normolipidemic adult volunteers was collected to evaluate three different solvent extraction protocols, including the classical Folch method, the methanol/tert-butyl methyl ether (MTBE) (Matyash) method, and a recent single-phase methanol/1-butanol (Alshehry) method. Extracted lipids were analyzed using liquid chromatography mass spectrometry (LC-MS) in positive and negative ion mode. Overall, more than 500 different lipids were identified in positive and negative ion mode combined. Our data show that the single phase Alshehry method was as effective as the Folch and Matyash methods in extracting most lipid classes and was more effective in extraction of polar lipids. Normalized peak areas of the Alshehry method were highly and positively correlated with both the Folch and Matyash methods (*r*^2^ = 0.99 and 0.97, respectively). Within- and between- subject correlations were *r* = 0.99 and 0.96, respectively. Median intra-assay coefficient of variation (CV%) in positive mode was 14.1, 15.1, and 21.8 for the Alshehry, Folch and Matyash methods, respectively. Median Alshehry inter-assay CV (collected over 5 separate days) was 14.4%. In conclusion, the novel Alshehry method was at least as good as, if not better than the established biphasic extraction methods in detecting a wide range of lipid classes, using as little as 10 μL of plasma, and was highly reproducible, safer and more environmentally-friendly as it doesn't require chloroform.

## Introduction

It is well-documented that lipids have multiple structural and functional roles, including signaling (1, 2), maintenance of membrane structure (3, 4), myelin sheath formation (5, 6), neurotransmission (7), and protein interactions in both plasma and organs including the brain (8, 9). Physiological processes such as synaptic and mitochondrial function, and lipid raft formation are critically dependent on lipid composition (10–12). By contrast, lipid by-products, particularly metabolites of arachidonic acid and lipid products of oxidative stress are drivers of inflammation (13). Consequently, the plasma lipidome has a considerable impact on the cellular lipid environment, vasculature function, and inflammatory and oxidative processes. The plasma lipid profile therefore also represents a lifestyle modifiable factor which can play a decisive role in the health state and maintenance of cognitive function during aging (14).

The field of lipidomics is constantly expanding, enabling high throughput analysis of lipid analytes in crude extracts for the study of health and disease. Recent advances in mass spectrometry have resulted in greater sensitivity, increased mass accuracy and faster scan speeds (15, 16). This has enabled greater sensitivity and better characterization of lipid changes in bodily fluids, cells, and tissue extracts, leading to renewed understanding of the role of different lipid classes in the pathobiology of diseases. Lipidomics has recently been applied to the study of dementia, where brain, CSF and plasma lipids have been identified as potential diagnostic and prognostic biomarkers for Alzheimer's disease (17, 18), frontotemporal dementia (19), as well as other common neuropsychiatric disorders, such as schizophrenia and bipolar disorder (20, 21).

The future of lipidomics lies in clinical application. As such, blood-based biomarkers are ideal since venepuncture is already routinely used in the clinic and is less invasive compared to CSF extraction via lumbar puncture. Additionally, it is much simpler to take repeat measurements of blood which may be useful for longitudinal analysis. We will discuss further the relationship of centrally derived biological signals with the periphery and vice versa in this paper. Although interest in lipidomics especially in the study of health and disease is expanding, there is still much room for improvement in lipidomic methodologies for research and clinical use. Since lipidomics often involves the study of 100 to 1,000 of individual biological samples within a single experiment, any improvement to the cost or efficiency of sample processing could have a considerable impact on streamlining plasma lipidomics research.

One key area in which improvement is possible is in the extraction process, whereby total lipids within plasma, tissue, and other biological extracts are isolated prior to further analysis. Solvent-based extraction systems must efficiently extract lipids present in biological samples while minimizing bias, lipid degradation/oxidation, or contamination from non-lipid components, such as sugars, peptides and amino acids. Therefore, effective and reliable identification and profiling of lipids in plasma is dependent on the efficiency of the lipid extraction protocol (22). The traditional Folch method (2:1 chloroform/methanol) (23), developed in 1957 is still used as a benchmark extraction process in many laboratories for a wide range of biological fluids, including, blood, tears, urine, saliva, cerebrospinal fluid, human milk, bronchoalveolar lavage fluid and sperm (22). The Folch method is based on the partitioning of lipids in a biphasic mixture of chloroform and methanol. Methanol disrupts hydrogen bonds between lipids and protein following addition of an organic solvent such as chloroform.

Alternative methods, such as the Matyash (tert-butyl methyl ether (MTBE)/methanol) (24), have gained popularity, in particular for the extraction of sphingolipids. Replacement of chloroform with MTBE demonstrated similar extraction of major lipid classes (22, 24). Recently, a single phase (1:1 1-butanol/methanol) extraction developed by Alshehry et al. (25, 26) has also been published. Both newer methods eliminate the need to draw lipids from the lower phase, thereby saving time compared to the Folch method, and are safer and more environmentally friendly as they do not use toxic chloroform. Moreover, the Alshehry method does not involve any biphasic solvent separation and could therefore be even more convenient, and potentially yield better lipid recoveries, than the Matyash method. The Matyash method has already been shown by independent laboratories to be as good, if not better, at extracting lipids as the Folch method (22, 24, 27). A recent comparative study of multiphasic methods has been published (28), however the single phase Alshehry method was only recently published and has not been compared with established biphasic methods.

**Figure 1:**
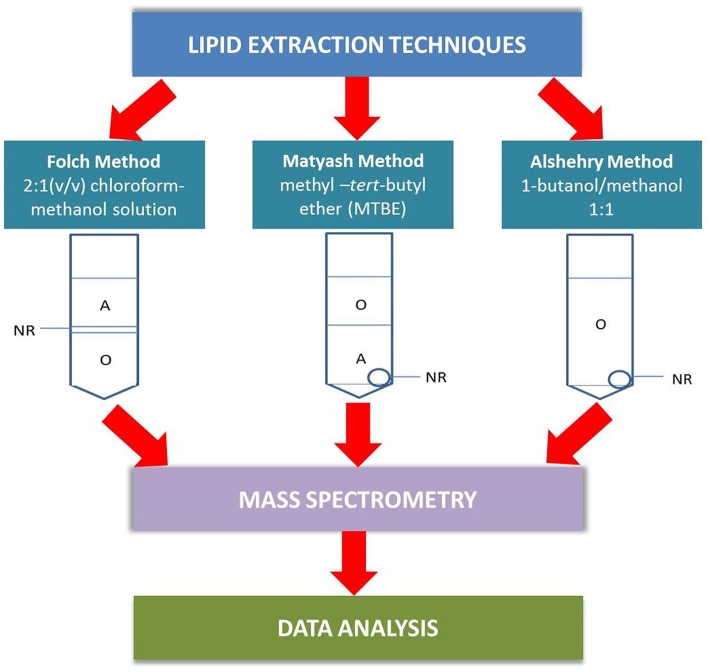
A schematic outlining the differences between the plasma extraction methods in the analytical pathway. In the Folch method, the lipid-containing organic phase (O) lies beneath a non-extractable residue (NR) and must be collected by passing a micropipette through the aqueous phase (A) and NR. The Matyash MTBE extraction simplifies the extraction process by having the lipids collected in the upper phase, though care must be taken not to take up the aqueous phase. The Alshehry 1-butanol/methanol extraction collects all lipids within a single-phase supernatant, with NR as a white pellet at the bottom of the tube.

In this paper, we examine the performance of the single-phase Alshehry extraction system and compare the results against the two biphasic extraction methods, the Matyash method and the traditional Folch method (Figure 1) in terms of coverage of lipids extracted and reproducibility from pooled plasma of healthy volunteers. Liquid chromatography mass spectrometry (LC-MS/MS) was used to identify the lipids extracted.

## Methods

### Reagents and Chemicals

HPLC grade methanol was purchased from Thermo Fisher Scientific (Sydney, Australia). Analytical reagent grade 1-butanol was purchased from Asia Pacific Specialty Chemicals (APS) (Sydney, Australia). Other high grade solvents and reagents were used, including MTBE (Sigma Aldrich, Israel), chloroform (Honeywell, USA), acetonitrile (Honeywell, Korea), formic acid (Chem-supply, Australia), ammonium formate (Honeywell, Germany). Isopropanol was of LC-MS grade (Honeywell, Michigan, USA). SPLASH Lipidomix stable isotope labeled internal standards were purchased from Avanti (Alabaster, Alabama, USA). All other reagents were analytical reagent grade. MilliQ water was used for buffer preparation and had a minimum resistivity of 18mΩ.

### Internal Standards

Internal standards were purchased from *Avanti* (Splash Lipidomix, Alabaster, United States) and included phosphatidylcholine 15:0–18:1(d7) PC, phosphatidylethanolamine 15:0–18:1(d7) PE, phosphatidylserine 15:0–18:1(d7) PS, phosphatidylglycerol 15:0–18:1(d7) PG, phosphatidylinositol 15:0–18:1(d7), PI, phosphatidic acid 15:0–18:1(d7) PA, lysophosphatidylcholine 18:1(d7) LPC, lysophosphatidylethanolamine 18:1(d7) LPE, cholesteryl ester 18:1(d7) CE, monoacylglycerol 18:1(d7) MG, diacylglycerol 15:0–18:1(d7) DG, triacylglycerol 15:0–18:1(d7)-15:0 TG, sphingomyelin 18:1(d9) SM and Cholesterol (d7). The Splash Lipidomix standards are deuterium labeled and not present endogenously, but with concentrations similar to physiological plasma concentrations of lipids for each class and thus are ideal for use as internal standards. A constant amount of internal standards was added to all samples and controls prior to lipid extraction (10 μL of a 1:10 dilution of SPLASH Lipidomix to 10 μL neat plasma) for normalization of raw peak areas and to correct for differences in extraction and ionization efficiencies and matrix effects (29).

### Blood Collection

Our study utilized fasting EDTA plasma collected from 10 cognitively “healthy” subjects aged between 65 and 85 years with informed consent, as part of a larger population-based study known as Sydney Memory and Aging Study (MAS) (30, 31). Subjects did not have any forms of mild cognitive impairment or dementia, as assessed by a panel of neuropsychiatrists according to consensus diagnosis criteria (30, 32), All subjects had mini-mental state examination (MMSE) scores >24 (33) and no history of cardiovascular complications or diabetes mellitus. Plasma taken from the 10 subjects (five males and five females) were then pooled and aliquoted (10 μL). Extractions from 10 pooled plasma aliquots were used to assess the intra-assay coefficient of variation (CV%) of each extraction method detailed below, while extractions taken on 5 separate days were used to assess inter-assay CV% of the Alshehry method. Blood collection, processing and storage were performed under strict conditions to minimize preanalytical variability (18, 34), which included restricting plasma collection and aliquot to freezer time to a maximum of 2 h, using EDTA plasma, and minimizing the number of freeze-thaw cycles. In particular, freezer storage of plasma and extracted lipids at −80°C prior to analysis and limiting the number of freeze thaw cycles, is now part of international standardization guidelines in order to maximize stability of lipids over prolonged periods of time (18, 35, 36).

The study was approved by the Ethics Committees of the University of New South Wales and the South Eastern Sydney and Illawarra Area Health Service (ethics approval HC12313 and HC14327, respectively). All work involving human subjects conformed to the principles of the Declaration of Helsinki of the World Medical Association.

### Lipid Extraction Protocols

#### Single Phase 1-Butanol/Methanol 1:1 (v/v) Alshehry Extraction Method

Lipids were extracted as previously described (25, 26). Briefly, we added 10 μL of internal lipid standards (*Avanti* SPLASH Lipidomix) that had been first diluted 1:10 in 1-butanol/methanol, to 10 μL aliquots of plasma in 0.5 mL polypropylene tubes (Eppendorf). 1-Butanol/methanol (100 mL, 1:1 v/v) containing 5 mM ammonium formate was then added to the sample, vortexed (10 s), then sonicated (1 h). Tubes were centrifuged (13,000 ×g, 10 min) and the supernatant removed into a clean polypropylene tube. A further 100 μl of 1-butanol/methanol (1:1 v/v) was added to the pellet to re-extract any remaining lipids. The combined supernatant was evaporated by vacuum centrifugation and stored at −80° C prior to analysis by LC-ESI MS/MS.

#### Traditional Biphasic 2:1 Chloroform/Methanol (v/v) Folch Extraction Method

Lipids were extracted as previously described (23), but using a 20-fold scale down of volumes used in order to produce comparable results against the same volume of plasma as used in the Alshehry method. Briefly, 10 μL aliquots of plasma and 10 μL of internal lipid standards (*Avanti* SPLASH Lipidomix) that had been first diluted 1:10 in 1-butanol/methanol were added to 160 μl of ice-cold methanol followed by addition of 320 μl of ice-cold chloroform and vortexed (10 s), then sonicated (1 h). Tubes were centrifuged at 10,000 ×g (10 min). The upper aqueous phase was transferred into a fresh polypropylene tube and re-extracted by addition of 250 μl ice-cold chloroform/methanol as described above. The upper phase was discarded, and the organic phase combined from both extractions, which was then evaporated by vacuum centrifugation and stored at −80°C prior to analysis by LC-ESI MS/MS.

#### Methanol-Tert-Butyl Methyl Ether Matyash Extraction Method

Lipids were extracted as previously described (24) with a 20-fold scale down of volumes; 10 μL aliquots of plasma and 10 μL of internal lipid standards (*Avanti* SPLASH Lipidomix) that had been first diluted 1:10 in 1-butanol/methanol were added to 400 μl of ice-cold methanol followed by addition of 500 μl of MTBE, vortexed (10 s), then sonicated (1 h). Afterwards, 500 μl of MilliQ water was added to induce phase-separation. Tubes were centrifuged (10,000 ×g, 10 min). The upper aqueous phase was transferred into a clean polypropylene tube and re-extracted by addition of 200 μl MTBE as described above. The upper phase was discarded, and the organic phase was evaporated by vacuum centrifugation and stored at −80° C prior to analysis.

### LC-MS Analysis

Extracted lipid samples and lipid internal standards (*Avanti* SPLASH Lipidomix) were removed from the −80° C freezer and resuspended in 100 μl of 1-butanol/methanol (1:1 v/v) containing 5 mM ammonium formate and transferred into Chromacol autosampler vials containing a 300 μl glass insert. Lipid analysis was performed by LC ESI-MS/MS (Dionex LC system in-line to a Thermo QExactive Plus Orbitrap mass spectrometer; ThermoFisher Scientific; Waltham, Massachusetts). A Waters ACQUITY UPLC CSHTM C18 1.7 μm, 2.1 × 100 mm column was used at a flow rate of 260 μL/min, using the following gradient: 32 to 100% solvent B over 25 min, a return to 32% B and finally isocratic 32% B (5 min) prior to the next injection. Solvents A and B consisted of acetonitrile: MilliQ water (6:4 v/v) and isopropanol: acetonitrile (9:1 v/v) respectively, both containing 10 mM ammonium formate and 0.1% formic acid. The first 3 min of eluent, containing sample salts, was diverted to waste. Product ion scans in positive and negative ion mode were performed on each sample, to maximize numbers of lipid species identified. Sampling order was randomized prior to analysis. LC-MS grade isopropanol was used to minimize intensity of the background signal.

### Alignment and Peak Detection Using LipidSearch 4.2.2

The raw data was aligned, and chromatographic peaks selected using LipidSearch software version 4.2.2 (Thermo Scientific, Tokyo, Japan), the database which now includes masses of the stable isotope labeled SPLASH Lipidomix internal standards. We performed search on raw files using the databases “General” and “labeled standards.” For peak detection, recalc isotope was set to “ON,” RT interval = 0.0 min. We used product search for LC-MS method and the precursor and product tolerances were set at 5.0 and 8.0 ppm, respectively. The intensity threshold was 1% parent ion, and the m-score threshold was set to 2.0. For quantitation, m/z tolerance was set at −5.0 to 5.0 ppm, and the retention time range was set at −0.5 to 0.5 min. The m-score threshold was 5.0, and all lipid classes were selected for inclusion. Ion adducts included +H, +NH4 for positive ion mode and –H, +HCOO for negative ion mode.

### Data Analysis

The results from LipidSearch 4.2.2 were exported as a.csv file and opened in Excel software for further data processing and analysis. Peak areas of lipids in samples were normalized by dividing with peak area of the corresponding internal standard for that lipid class (i.e., peak area ratios). Correlations between normalized peak areas from the three extraction methods were calculated using the SPSS statistics software. The intra-assay coefficient of variation (CV) was calculated by dividing the standard deviation of the normalized abundances of each lipid (for each method) by the mean across lipid species. Lipid ion identifications were filtered using the LipidSearch parameters rej = 0 and average peak quality>0.75. Recovery was calculated as the average raw intensity of internal standards spiked before extraction and divided by the average raw intensity of internal standards spiked post extraction. The inter-assay CV% for the Alshehry method was also determined by comparing measurements from extractions taken across 5 separate days within a month.

## Results

### Concordance Between Methods

**Figure 2:**
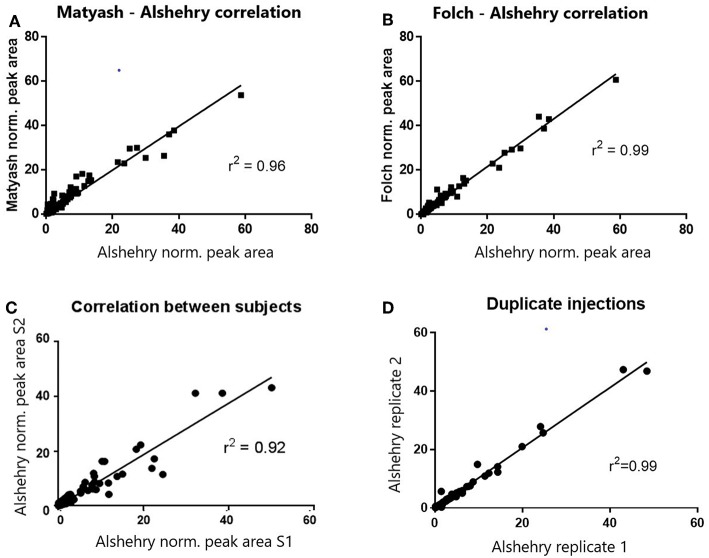
Correlations of normalized peak areas between methods **(A,B)**, subjects **(C)** and runs **(D)**. Normalized peak areas of the Alshehry method were highly and positively correlated with both the Folch and Matyash methods. Within-subject correlation across separate runs was also high, though between- subject correlation was lower.

When we compared the coverage of the different lipid classes based on extracted lipid internal standard peak areas, normalized peak areas in both positive and negative modes were highly and significantly correlated with the biphasic Matyash and Folch methods (*r* = 0.97 and 0.99 respectively, *p* < 0.0001, Figures 2A,B). This concordance was also reflected when comparing lipid measurements between duplicate extractions of plasma lipids prepared using the Alshehry protocol (*r*^2^ = 0.995), and in two different subjects, which is indicative of high reproducibility (Figures 2C,D). Additionally, Bland-Altman analyses revealed that the Alshehry method largely agreed with the Folch and Matyash methods (bias ratio = 1.09 and 1.247, respectively), except more for some lowly abundant peak areas, which were higher using the Alshehry method. All three methods produced similar number of LipidSearch molecular ion IDs prior to filtering (381, 400, and 390 IDs in positive ion mode for the Matyash, Folch and Alshehry methods respectively, and 122, 127, and 119 IDs in negative ion mode, similarly).

### Comparison of Lipid Abundances

**Figure 3:**
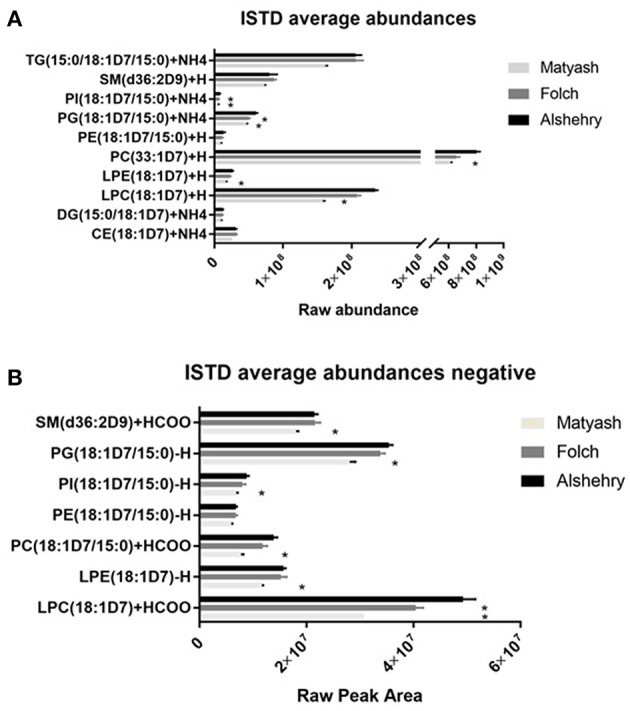
ISTD raw abundances across the three extraction methods in **(A)** positive ion mode and **(B)** negative ion mode. The Alshehry method was as effective as the Folch and Matyash method in extracting most lipid classes and was more effective in extraction of more polar lipids (LPC, LPE and PI). Error bars represent SE (*p < 0.05 relative to single phase method). CE, cholesterol ester; D5DG, deuterated diacylglycerol; DG, diacylglycerol; LPC, lysophosphatidylcholine; LPE, lysophosphatidylethanolamine; PC, phosphatidylcholine; PE, phosphatidylethanolamine; PG, phosphatidylglycerol, PI, phosphatidylinositol, PS, phosphatidylserine, SM, sphingomyelin; TG, triacylglycerol.

One-way ANOVA was used to test whether the raw peak areas for internal standards using the Alshehry method were significantly different from the Matyash or Folch methods. There were no significant differences in peak areas between extraction methods for DG, PE and SM species (*p* > 0.05). Peak areas of LPC, LPE, PI, PC and PG were higher in the Alshehry method than the Matyash method (*p* < 0.05, Tukey's *post-hoc* test), while the peak area of PG and PI were also greater in the Alshehry method relative to the Folch extraction. The Alshehry method was more efficient in extracting the highly polar lipids, such as LPC and LPE internal standard relative to the Matyash method (*p* < 0.05, one-way ANOVA, Tukey's *post-hoc* test) (Figure 3A).

Interestingly, TG was significantly higher in the Folch method compared to Matyash (*p* < 0.05, Tukey's *post-hoc* test), but yielded comparable, or slightly higher peak areas compared to Alshehry method, which did not reach significance (*p* = 0.08). Similarly, Alshehry peak areas of internal standards were larger than or equal to the Folch, and larger than the Matyash method in negative ion mode (Figure 3B, all *p* < 0.05, Tukey's *post-hoc* test). Overall, the Alshehry method produces similar, if not greater yields than either the Folch or Matyash method.

### Recovery of Internal Standards

**Figure 4:**
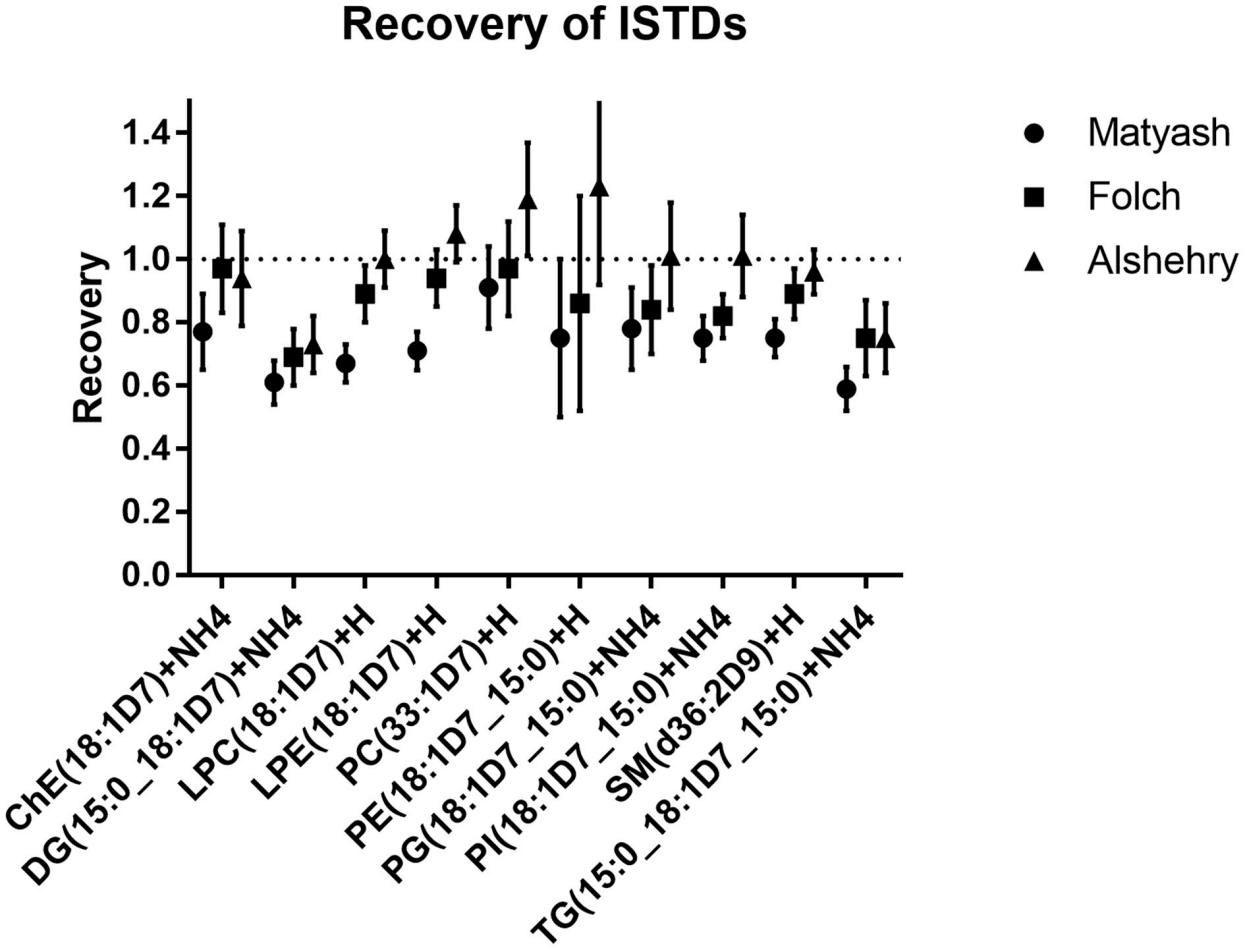
Recoveries of internal standards for extraction methods. The values represent ratios of peak areas ± SD (error bars) relative to unextracted internal standards (i.e., spiked after solvents were added).

Recoveries for each method were assessed by comparing the ratio of peak areas of internal standards spiked prior to and after extraction (Figure 4). The average recoveries were 99, 86, and 73% for the Alshehry, Folch and Matyash extractions, respectively. The highest recoveries for the Alshehry method were achieved with the phospholipids (>95%) and lowest for the less polar TG, DG lipids (<80%).

### Reproducibility of Methods

**Figure 5:**
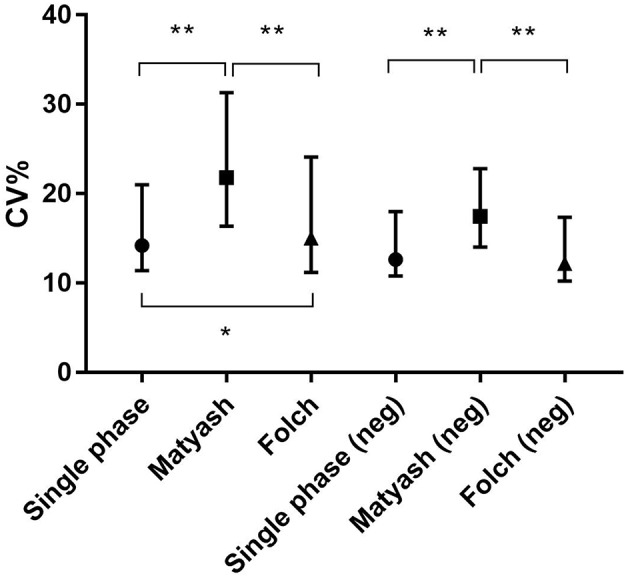
Intra-assay CV was lower in single phase extraction relative to the Folch and Matyash methods for all lipids in positive ion mode and negative ion mode. Error bars represent median ± interquartile range. *p < 0.05, **p < 0.001, Mann-Whitney *U*-test.

**Figure 6:**
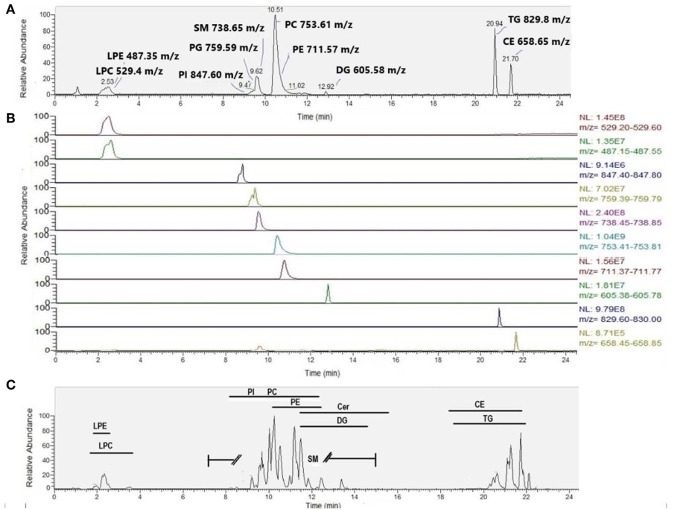
Typical chromatogram featuring **(A)** SPLASH Lipidomix internal standards in positive ion mode (total ion chromatogram), **(B)** SPLASH Lipidomix internal standards (extracted ion chromatograms for each standard narrowed to ±0.2 Da of the theoretical *m/z*), presented from top to bottom in order of increasing retention time, and **(C)** corresponding plasma sample in positive ion mode. Typical retention time ranges of elution (minutes) are provided for various lipid classes.

We also compared intra-assay coefficients of variation (Figure 5), defined as the standard deviation of the peak areas across several samples in a single experimental batch divided by the mean of the peak area, expressed as a percentage. A lower CV% then corresponds to consistency of values obtained from extraction of technical replicates. The median intra-assay (repeat injections within a single experiment) and inter-assay (injections from samples processed across separate days) coefficients of variation (CV%) for all lipids in positive ion mode originally reported by Alshehry were 12 and 14% respectively (37). Here, we report median intra-assay and inter-assay CV of 14.2 and 14.4%, respectively for the Alshehry extraction, which is consistent with this previous report. The median intra-assay CV% we obtained using the Alshehry method was lower compared to that obtained for the Matyash and Folch methods (21.8 and 15.1% respectively, *p* < 0.0001 and *p* = 0.014 respectively, Mann-Whitney *U* test). Seventy-five percent of lipids in the Alshehry method had a CV of 21% or lower, and the median CV for internal standards was 8.5%. Median CV for lipids in negative mode were 12.7, 17.5, and 12.2% for the Alshehry, Matyash and Folch recipes respectively (*p* < 0.0001 for Folch and Alshehry relative to Matyash, Mann-Whitney *U*-test). The comparative data collected in this experiment is summarized in Table 1. Typical representative total ion chromatograms of internal standards (Figure 6A), alongside extracted ion chromatograms for each standard (Figure 6B), as well as the total ion chromatogram for plasma lipids (Figure 6C) are shown.

**Table 1:** Results summary.

	**Matyash**	**Folch**	**Alshehry**
Volume of plasma per sample (this study)	10 μL	10 μL	10 μL
Volume of plasma per sample (original)	200 μL	200 μL	10 μL
Volume of solvents per sample (this study)	900 μL	480 μL	100 μL
Volume of solvents per sample (original)	18,000 μL	9,600 μL	-
Numbers of individual lipids in positive ion mode	381	400	390
Numbers of individual lipids in negative ion mode	122	127	119
Median intra-assay CV% in positive ion mode	21.8	15.1	14.1
Median intra-assay CV% in negative ion mode	17.5	12.2	12.7
Average total recovery %	73	86	99

## Discussion

In the original study by Alshehry et al. (25) the single phase 1-butanol/methanol 1:1 (v/v) method was reported to be as good an extraction as similar single phase extraction protocols with lipid measurements (based on peak areas normalized against internal standards). In particular, results between the Alshehry method and a single phase 2:1 methanol/chloroform extraction method were strongly correlated (*r*^2^ = 0.976). In the present study, both the Matyash and Folch methods were also strongly correlated with Alshehry method (*r* = 0.97 and 0.99, respectively) which suggests strong agreeance between the methods, also supported by Bland-Altman bias ratios. Overall, this suggests that methods correspond well, though the Alshehry method may be superior in detection of more lowly abundant lipid species. Number of extracted lipids were also similar between the methods indicating similar coverage of lipids for all three extractions.

Although most lipids classes were extracted similarly between methods, certain lipid classes were more abundantly extracted using the Alshehry method as opposed to the Matyash or Folch methods. This included the lysophospholipids LPC and LPE, as well as some glycerophospholipids containing a polar head group, such as PC, PI and PG. This is consistent with reports that the Alshehry method is more amenable to extraction of highly polar lipids compared to other methods (25), since the hydrophilic phases are discarded in these biphasic methods. A previous pilot analysis also showed markedly greater extraction of the highly polar lipid standards, phosphatidylserine, PS(17:0/17:0) and sphingosine phosphate, SoP(d17:1) in the Alshehry method compared to either the Matyash or Folch methods. The ability of the Alshehry method to capture more polar lipids may be particularly useful in analysis of neuropsychiatric disorders and dementia where these lipids have been implicated in these conditions (17, 38, 39).

Although the Alshehry recipe is ideal for extracting more polar lipids, the method is adaptable for extraction of other lipid classes. For example, a similar study of lipid extraction in cow's milk applied a modified version of the Alshehry single phase method in order to extract higher levels of non-polar lipids (40). This modified version used a single phase mixture of 1-butanol/methanol/chloroform (3:5:4 v:v:v) in order to broaden the coverage of highly lipophilic species, especially triglycerides. Other similar single-phase extraction procedures using different solvent ratios have been described for lipid extraction, such as a 3:1 butanol/methanol extraction for plasma (41) and animal tissue (42), as well as a methanol/MTBE/chloroform (1.33/1/1 v:v:v) extraction (43, 44). All these cases, report comparable, if not greater yields against the Matyash and Folch methods suggesting good feasibility of single-phase extraction methods against traditional biphasic methods, as well as adaptability to capture a wide range of lipids of interest.

Overall, there was strong recovery for a large majority of lipids with the single-phase method, at 99%, especially for polar lipids. The high recovery of the Alshehry method near 100% is consistent with the fact that this method does not experience the risk of dripping losses that occurs with the other two biphasic methods as the bulk of lipids are present in a single phase to handle and readily removable for lipid analysis.

The less than optimal recovery (73%) for the Matyash method compared to previously reported is likely related to the relatively low volume of solvent used during the 20-fold volume scale down procedure, as well as the tendency of MTBE to evaporate quickly, affecting extraction reproducibility (41), which is also reflected in the higher CV% (see below).

Analysis of reproducibility based on CV% revealed the Alshehry method had a median CV% of 14.2%, lower than or equal to the Folch method, and even better than the Matyash method (at 15.1 and 21.8% respectively, in positive ion mode). Thus, the Alshehry protocol produces reliable lipid measurements over technical replicates within experimental batches as well as, or even better than the established Folch and Matyash approaches, which is expected considering reduced dripping losses in a monophasic extraction. Additionally, the inter-assay CV% is very similar to that of the intra-assay CV% for the Alshehry method which suggests that the method produces reliable measurements across experimental batches and any additional variation to this method due to batch differences would be minimal.

Furthermore, the single phase Alshehry method is advantageous in that lipids are drawn into a single phase and can be easily removed from the sample as supernatant without picking up non-extractable matrix. This makes the method easier and potentially faster compared to conventional two-phase solvent partition systems where there is also an increased risk of contamination from unwanted analytes, and the risk of losses during transfer between phases. Additionally, the method can be applied to as little as 10 μl of plasma, and using just 100 μl of solvent per sample, and therefore greatly minimizes the amount of sample and solvent required for extraction. By contrast, the original Matyash extraction reports extractions using 200 μl of plasma, and requires 900 μl of MTBE and methanol combined pre-scale down, which translates to 18 ml of solvent required per plasma sample in the original Matyash method. Considering the broad range of lipids and reduced losses reflected in the high degree of recovery and reproducibility obtainable using a relatively small volume of sample and solvent in the Alshehry method, this represents a strong advantage of the method over existing biphasic methods. The volume savings can be particularly important for high value plasma samples from expensive clinical and population-based studies, since such samples are a finite resource and conservative usage is necessary, to maximize potential experiments and makes extraction of many samples much simpler to handle.

This new method has already been applied for lipid extraction of plasma from large populations for the study of health and disease (37, 45). Nevertheless, we highlight one important limitation of the Alshehry method as reported by the original authors (25): due to the monophasic nature of extraction, ionic contaminants are not readily removed during extraction, leading to an increased risk of ion suppression effects. The Alshehry method is therefore unsuitable for shotgun/direct infusion lipidomics techniques, but is suitable for use with reverse phase LC-ESI MS/MS.

It needs acknowledging that in context of neuropsychiatric disorders and dementia, it is important to establish the connection between putative changes in peripheral signals with that of signals generated by the central nervous system (CNS). Despite all the advantages associated with peripheral biomarkers, a limitation of plasma-based lipidomics research is that the blood brain barrier (BBB) restricts passage of metabolites between the CSF and blood compartments. Therefore, there is not always a strong correspondence between blood and centrally derived metabolites. Nevertheless, many CSF metabolites are selectively reabsorbed back into the vascular system, and essential lipids such as polyunsaturated fatty acids are transported to the brain from blood as free fatty acids (46) or as part of phospholipids, such as lysophosphatidylcholine (47), a lipid class well-detected using the novel method. The BBB may have increased permeability in response to inflammation or disease states (48, 49). Moreover, there is substantial overlap in metabolic changes identified between matched CSF and plasma (50, 51), and some blood lipids may be correlated with CSF tau and amyloid load (52), as well as other imaging measures such as brain atrophy (53).

Other studies have also identified relationships between plasma/serum lipids, apolipoproteins and cognition (54, 55). A recent study identified differential impacts of apolipoprotein E in the CNS and periphery, finding deficits in apolipoprotein E in the CNS induced synaptic loss, whereas a deficit in the periphery lead to impaired cognition (56), though synaptic loss could partially be rescued by increasing plasma apolipoprotein E levels. The potential of plasma lipids to predict memory performance and cognitive decline (or protection) several years in advance (57–59) presents evidence that these lipids may have a substantial influence on the CNS, or that plasma lipids may reflect some underlying pathological process occurring in the CNS. Many of the metabolites reported in the above studies are robustly extracted using the novel Alshehry method, such as sphingolipids, PC, PE and their corresponding lysophospholipids, and have also similarly been found to be altered in *post-mortem* brain tissue or CSF in patients with Alzheimer's disease (38, 50, 60). We have previously reviewed plasma lipids thought to be altered in Alzheimer's disease (18), which includes plasmalogen phospholipids, sphingomyelins and ceramides. Further, a recent untargeted lipidomics study in plasma found substantial differences in the lipidomic profile between healthy controls, Alzheimer's disease and behavioral variant frontotemporal dementia (bvFTD) (19). Of note, the authors were able to confirm hypertriglyceridemia in bvFTD at the lipid species level, and also detected altered levels of dietary lipids thought to characterize binge eating behaviors among these patients. This study, among others demonstrates the utility of exploring plasma lipids as potential biomarkers for neuropsychiatric and neurological disorders. Even with such a wealth of studies, independent validation and replication remains a high priority concern in the lipid biomarker research community (18). Further, the natural variation in lipids within and between individuals independent of disease is still being characterized (45, 61, 62), which is an important factor to consider before applying comparative lipidomics in disease. Although this pilot study does not focus on inter-individual differences in lipids, we have recently applied this particular method in a larger study of 100 individuals to identify the natural variation of the plasma lipidome in cognitively healthy individuals by age, sex and BMI (63). For future studies, we intend to investigate the influence of *APOE* genotype on the plasma lipidome, as well as identify lipidomic changes associated with vascular dementia. We believe that a consistent and simple method for plasma lipid extraction, such as explored in this paper, will go a long way toward expanding blood lipidomic studies, give due insight into neurological processes and minimize some of the variance in literature of reported biomarkers inherent in extraction methodology.

## Conclusion

Overall, we confirm the findings of the originally published Alshehry single-phase protocol, but also show that it is good as, if not better than the commonly used biphasic Matyash and Folch methods in the general extraction of plasma lipids, based on its ability to cover a broad range of lipid classes, including more polar lipids that may not be extracted readily using the Folch recipe. The Alshehry method also provides reliable and reproducible measurements when lipids are analyzed by LC-MS/MS above that of the Folch and Matyash methods. The single-phase method offers a safe, environmentally friendly (chloroform-free) and economical method for extraction of plasma lipids with high (nearly 100%) recovery that is a good substitute for the traditional approaches. Notably, all methods produce highly correlated results, indicating that reliable data can be obtained with all these protocols. However, the simplicity involved in having most lipids extractable in a single phase and low plasma and solvent volumes required by this Alshehry method may be particularly useful where sample volumes are limited, for projects where high sample numbers need to be assayed for lipidomic analysis, or where plasma needs to be conserved to maximize experiments.

## Data Availability

The datasets generated for this study are available on request to the corresponding author.

## Ethics Statement

The study was approved by the Ethics Committees of the University of New South Wales and the South Eastern Sydney and Illawarra Area Health Service (ethics approval HC12313 and HC14327, respectively). All work involving human subjects conformed to the principles of the Declaration of Helsinki of the World Medical Association and both informed and written consent was obtained.

## Author Contributions

MW, NB, and AP wrote and drafted the manuscript. NB, RP, AP, and PS conceived of the project. MW performed the experiments and analyzed the data. RP provided technical expertise and training for use of the QExactive Plus mass spectrometer and LipidSearch software.

### Conflict of Interest Statement

The authors declare that the research was conducted in the absence of any commercial or financial relationships that could be construed as a potential conflict of interest.
